# MiR-377-3p inhibits atherosclerosis-associated vascular smooth muscle cell proliferation and migration via targeting neuropilin2

**DOI:** 10.1042/BSR20193425

**Published:** 2020-06-15

**Authors:** Haijun Wang, Zheng Wei, Hulun Li, Yinghui Guan, Zhiyang Han, Hengzhen Wang, Bing Liu

**Affiliations:** 1 Department of Vascular Surgery, The First Affiliated Hospital of Harbin Medical University, Harbin 150001, People’s Republic of China; 2 Department of Neurology, Harbin Medical University, Harbin 150001, People’s Republic of China

**Keywords:** Atherosclerosis, MicroRNA-377-3p, Migration, Neuropilin2, Proliferation, Vascular smooth muscle cells

## Abstract

Vascular smooth muscle cell (VSMC) proliferation and migration are vital to atherosclerosis (AS) development and plaque rupture. MicroRNA-377-3p (miR-377-3p) has been reported to inhibit AS in apolipoprotein E knockout (ApoE^−/−^) mice. Herein, the mechanism underlying the effect of miR-377-3p on alleviating AS is explored. *In vivo* experiments, ApoE^−/−^ mice were fed with high-fat diet (HFD) to induce AS and treated with miR-377-3p agomir or negative control agomir (agomir-NC) on week 0, 2, 4, 6, 8, 10 after HFD feeding. MiR-377-3p was found to restore HFD-induced AS lesions and expressions of matrix metalloproteinase (MMP)-2, MMP-9, α-smooth muscle actin (α-actin) and calponin. In *in vitro* experiments, human VSMCs were tranfected with miR-377-3p agomir or agomir-NC, followed by treatment with oxidized low-density lipoprotein (ox-LDL). MiR-377-3p was observed to significantly inhibit ox-LDL-induced VSMC proliferation characterized by inhibited cell viability, expressions of proliferating cell nuclear antigen (PCNA), cyclin D1 and cyclin E and cell cycle transition from G_1_ to S phase accompanied with less 5-Ethynyl-2′-deoxyuridine (EdU)-positive cells. Furthermore, MiR-377-3p significantly inhibited ox-LDL-induced VSMC migration characterized by inhibited wound closure and decreased relative VSMC migration. Besides, neuropilin2 (NRP2) was verified as a target of miR-377-3p. MiR-377-3p was observed to inhibit NRP2 expressions *in vivo* and *in vitro*. Moreover, miR-377-3p significantly inhibited MMP-2 and MMP-9 expressions in human VSMCs. Additionally, miR-377-3p-induced inhibition of VSMC proliferation and migration could be attenuated by NRP2 overexpression. These results indicated that miR-377-3p inhibited VSMC proliferation and migration via targeting NRP2. The present study provides an underlying mechanism for miR-377-3p-based AS therapy.

## Introduction

Cardiovascular disease (CVD) is the underlying cause of all deaths in low- and middle-income countries [1]. Atherosclerosis (AS), a chronic disease, is the primary cause of CVD, including coronary heart disease (CHD), cerebral infarction and peripheral arterial disease (PAD). Disorder of lipid metabolism is a risk factor for AS [2]. AS lesion is characterized by the accumulation of lipid and fibrous elements in the intima of artery and the thickening and hardening of artery walls [3]. In spite of the improvement in living standards and therapeutic strategies, AS-induced CVD continues to be the principal cause of global deaths and accounts for approximately 31% of all deaths worldwide (WHO, 2017). As a consequence, exploring the underlying mechanism of AS therapy is of vital importance for AS treatment.

MicroRNAs (miRNAs), are endogenous, small non-coding RNA molecules. They base pair with the 3′ untranslated region (3′UTR) of target genes to negatively regulate the expression of target genes. At present, miRNAs have been found to be involved in a variety of biological processes such as the development of aging and diseases [4]. Accumulating evidence has suggested that miRNAs may be involved in AS development [5,6]. MiR-377, which is located in chromosome region 14q32, is emerged to suppress tumors [7], oxidative stress and inflammation in ischemic hearts [8] and glomerular podocyte injury [9], and promote cardiac regenerative ability in stem cells after ischemia–reperfusion injury [10]. A previous report has shown that miR-377 reduced triglyceride levels in plasma and significantly decreased the area of aortic lesions in apolipoprotein E knockout (ApoE^−/−^) models [11]. In addition, miR-377 can regulate inflammation and suppress the abnormal proliferation and migration of brain microvascular endothelial cells (ECs) [12]. Hence, it was concluded that miR-377 might attenuate the development of AS. Multiple types of cells participate in the pathogenesis of AS, such as vascular smooth muscle cells (VSMCs), ECs and macrophages [13]. The migration and proliferation of VSMCs are key elements in AS development, which can accelerate the pathological process of AS [14]. At present, the effect of miR-377 on inhibiting the migration and proliferation of brain microvascular ECs [12] and cancer cells [15] has been reported, whereas its effect on the migration and proliferation of VSMCs in AS is still unclear.

ApoE is well known as a suppressor for AS, which plays a key role in the transport of cholesterol and other lipids [16]. ApoE deletion accumulates cholesterol and promotes the development of AS plaques, which results in hypercholesterolemia and spontaneous AS [17]. At present, ApoE^−/−^ mice and high-fat diet (HFD) treatment have been widely used in many AS-related studies. Oxidized low-density lipoprotein (ox-LDL) is considered as a risk factor in AS [18]. In this study, HFD and ox-LDL treatment were used to induce AS *in vivo* and *in vitro*, respectively. The effect of miR-377-3p on AS development and ox-LDL-induced cell proliferation and migration was explored in AS mice and human VSMCs, respectively. Moreover, the underlying mechanism of miR-377-3p in regulating the proliferation and migration of human VSMCs was investigated.

## Materials and methods

### Animals

Six-week old male ApoE^−/−^ mice and wild-type mice were maintained under a 12-h light/12-h dark cycle with temperature 25 ± 1°C and humidity 45–55%, and free access to water and food. Animal experiments were performed in accordance with National Research Council (U.S.) Committee for the Update of the Guide for the Care and Use of Laboratory Animals, and were approved by the Ethics Committee of The First Affiliated Hospital of Harbin Medical University. The animals’ experiments were performed in the Animal Research Center of Harbin Medical University (Harbin, China).

The wild-type mice were fed with normal diet (control group). The ApoE^−/−^ mice were randomly divided into three groups: HFD group, HFD and miR-377-3p agomir group (HFD+agomir-377), and HFD and agomir negative control group (HFD+agomir-NC). HFD contains 16.6% fat, 10.6% sucrose and 1.3% cholesterol [19]. At week 0, 2, 4, 6, 8 10 after HFD feeding, HFD+agomir-377 group and HFD+agomir-NC group were treated with miR-377-3p agomir and agomir-NC by tail-vein injection (50 mg/kg), respectively. All mice were killed by intraperitoneal injection with Nembutal (200 mg/kg) at week 12 after HFD feeding. Subsequently, the aortas were excised from the killed mice, and were fixed and frozen for further analyses.

### Hematoxylin and Eosin staining and immunohistochemistry analysis

For Hematoxylin and Eosin (H&E) staining, fixed aorta tissues were successively dehydrated in 20 and 30% sucrose, embedded in O.C.T compound and then sectioned at 10 μm. The sections were stained with Hematoxylin (Solarbio, Beijing, China) and Eosin (Sangon, Shanghai, China) and observed under a microscope (40× and 200×, OLYMPUS, Tokyo, Japan). For immunohistochemistry (IHC) analysis, fixed samples were embedded in O.C.T compound, sectioned at 10 μm and fixed in pre-cooled acetone for 15 min. The sections were immersed in sodium citrate buffer for antigen retrieval and then incubated in 3% hydrogen peroxide for 15 min to eliminate endogenous peroxidase activity. Next, the sections were incubated in normal goat serum (Solarbio) for 15 min. Subsequently, the sections were incubated with α-SMA or calponin antibody (1:200 dilution, Proteintech) overnight at 4°C, followed by incubation with goat anti-rabbit IgG-HRP antibody (1:500 dilution, Thermo Fisher Scientific, Waltham, MA, U.S.A.) for 60 min at 37°C. The sections were stained with diaminobenzidine (DAB), counterstained with Hematoxylin and observed under a microscope (400×, OLYMPUS).

### Cell culture, transfection and treatment

Human VSMCs were purchased from iCell Bioscience (Shanghai, China) and cultured in Ham’s F-12K medium (Procell, Wuhan, China) containing 10% fetal bovine serum (FBS, BI, Kibbutz Beit Haemek, Israel), 0.05 mg/ml ascorbic acid, 0.01 mg/ml insulin, 0.01 mg/ml transferrin, 10 ng/ml sodium selenite, 0.03 mg/ml ECGS, 10 mmol/l HEPES and 10 mmol/l TES in 5% CO_2_ at 37°C. The miR-377-3p agomir or agomir-NC was mixed with Lipofectamine™ LTX and Plus Reagent (Invitrogen, Carlsbad, CA, U.S.A.) and transiently transfected into human VSMCs. For co-transfection, miR-377-3p agomir/agomi-NC and neuropilin2 (NRP2) overexpression vector were co-transfected into human VSMCs. After 4-h transfection, cells were treated with 50 mg/l ox-LDL (Union-Biology, Beijing, China) for 48 h.

### Cell counting kit-8 assay

Cell counting kit-8 (CCK-8) assay kits (Sigma, St. Louis, MO, U.S.A.) were used to measure cell viability. Cells were seeded in 96-well plates at a density of 4 × 10^3^ per well. After cell transfection and ox-LDL treatment, the medium was replaced with 100 μl complete medium and then 10 μl CCK-8 per well was added into plates. Subsequently, the absorbance was measured at 450 nm using an Absorbance Microplate Reader (BioTek, Winooski, VT, U.S.A.).

### Cell cycle distribution

Cell cycle analysis kits (Beyotime, Shanghai, China) were used to analyze cell cycle distribution according to the manufacturer’s instructions. Cells were seeded in six-well plates at a density of 4 × 10^5^ per well. After cell transfection and ox-LDL treatment, cells were fixed in 70% ice-cold ethanol at 4°C for 12 h and then stained with propidium iodide solution and RNase A at 37°C for 30 min. Cells were detected using a flow cytometer (NovoCyte, ACEA Bio, San Diego, CA, U.S.A.).

### 5-Ethynyl-2′-deoxyuridine staining

Cells at S phase were detected using Click-iT EdU Kits (KeyGEN, Nanjing, China) according to the manufacturer’s protocols. The 5-Ethynyl-2′-deoxyuridine (EdU)-positive and Hoechst 33342-positive cells were counted under a fluorescent microscope (400×, OLYMPUS).

### Wound healing assay

After cell transfection, cells were treated with 1 μg/ml mitomycin C for 1 h when cells reached confluence. Then a wound was scratched using a sterile 200 μl-pipette tip. Cells were observed under a microscope before and after ox-LDL treatment (100×, OLYMPUS).

### Transwell assay

Transwell chambers were placed in 24-well plates and the bottom chambers were immersed in 20% FBS. After cell transfection, cells were seeded into the top chamber at a density of 5 × 10^3^ per well. After ox-LDL treatment, migrated cells were fixed with 4% PFA and stained with 0.4% Crystal Violet. Cells were observed under a microscope (200×, OLYMPUS).

### Dual luciferase reporter assay

Cells were seeded into 12-well plates and starved for 1 h when cells reached 70% confluence. The wild-type (wt) or mutated (mut) 3′UTR fragments of NRP2 containing the predicted binding site of miR-377-3p was inserted into a reporter vector. Then the vector was co-transfected with miR-377-3p agomir or agomir-NC into human VSMCs. After 48-h cultivation, cells were harvested and relative luciferase activity was measured using dual luciferase reporter assay kits (Promega, Madison, WI, U.S.A.).

### Quantitative real-time polymerase chain reaction

Total RNA was extracted from aortas and human VSMCs using RNApure total RNA fast isolation kits (BioTeke, Beijing, China) according to the manufacturer’s protocols. The concentration of total RNA was quantified by a spectrophotometer (Thermo Fisher Scientific). Reverse transcription was performed to synthesize cDNA using reverse transcriptase M-MLV (Takara, Otsu, Japan) and oligo (dT)15 and random primers or specific miRNA RT primers. The quantitative real-time polymerase chain reaction (qRT-PCR) of miR-377-3p and NRP2 was performed with SYBR Green (BioTeke) and Taq HS Perfect Mix (Takara). U19 and β-actin were used to normalize miR-377-3p and NRP2 expression, respectively. The relative levels were presented as 2^−ΔΔ*C*_t_^. The primer sequences used in the present study were listed in Table 1.

**Table 1:** Primer sequences used for qRT-PCR in the present study

Gene	Forward (5′–3′)	Reverse (5′–3′)
*MiR-377-3p*	ATCACACAAAGGCAACTTTTGT	GGTGCAGGGTCCGAGGTAT
*Homo U19*	TGGAGTTGATCCTAGTCTGG	GTGCAGGGTCCGAGGTATTC
*Mus U19*	TGTGGAGTTGGTCCTGGTCT	GTGCAGGGTCCGAGGTATTC
*NRP2*	ATTCCAAAGATGCTGGCTAT	GAGGGTTGAAGTTGAGGACA
*β-actin*	CACTGTGCCCATCTACGAGG	TAATGTCACGCACGATTTCC

### Western blot analysis

Total protein was extracted by RIPA Lysis Buffer (Beyotime) containing 1% phenylmethanesulfonyl fluoride (Beyotime). After centrifugation, total protein content was quantified using a bicinchoninic acid (BCA) protein assay kit (Solarbio). The protein was separated by sodium dodecyl sulfate/polyacrylamide gel electrophoresis (SDS/PAGE) and transferred on to a polyvinylidene difluoride (PVDF) membrane (Thermo Fisher Scientific). After blocking with 5% BSA in TBST, the membranes were probed with primary antibodies overnight at 4°C. Then the membranes were incubated with secondary antibodies for 40 min at 37°C. The primary antibodies contained α-smooth muscle actin (α-SMA) antibody (1:500 dilution, CST, Danvers, MA, U.S.A.), calponin antibody (1:1000 dilution, CST), proliferating cell nuclear antigen (PCNA) antibody (1:500 dilution), cyclin D1 antibody (1:500 dilution, CST), cyclin E antibody (1:1000 dilution), NRP2 antibody (1:400 dilution, Bioss, Beijing, China), matrix metalloproteinase (MMP)-9 antibody (1:1000 dilution), MMP-2 antibody (1:500 dilution), β-actin (1:2000 dilution). The secondary antibodies contained goat anti-rabbit IgG-HRP and goat anti-mouse IgG-HRP (1:10000 dilution). Except for α-SMA antibody, calponin antibody, cyclin D1 antibody and NRP2 antibody, other antibodies were purchased from Proteintech (Wuhan, China). The gray values of blots were analyzed by Gel-Pro-Analyzer software (Media Cybernetics, Rockville, MD, U.S.A.). Data of target proteins were normalized to β-actin control.

### Statistical analysis

Results were reported as means values ± standard deviation (SD). Statistical analysis of cell viability was performed with two-way analysis of variance (ANOVA, Figure 2B) or unpaired *t* test (Figure 5D), that of data from two groups with unpaired *t* test and that of data from multiple groups with one-way ANOVA by GraphPad Prism 8.0.1 software.

## Results

### MiR-377-3p treatment attenuates aortic lesions in AS mice

**Figure 1:**
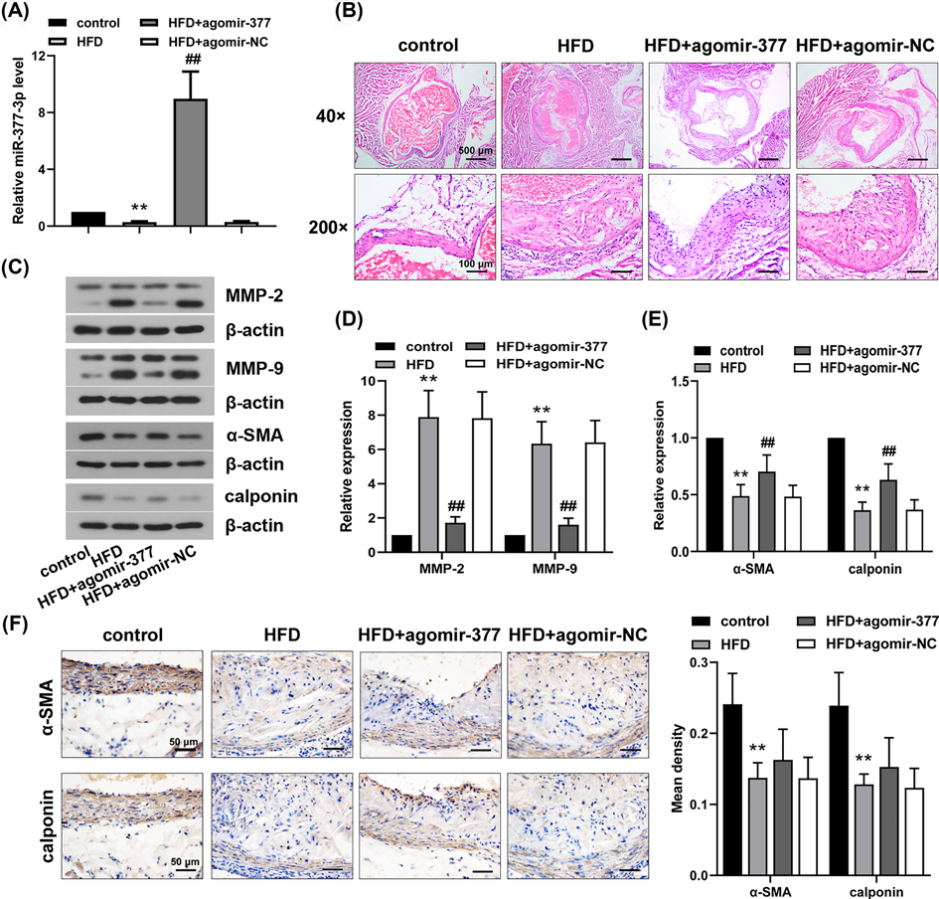
MiR-377-3p suppresses the progression of AS lesions in AS mice ApoE^−/−^ mice were fed with HFD for 12 weeks and injected with 50 mg/kg miR-377-3p agomir or agomir-NC at week 0, 2, 4, 6, 8 10 after HFD feeding. After 12 weeks, aortas were excised, fixed and frozen for further analyses. (**A**) MiR-377-3p mRNA levels in aortas using qRT-PCR. (**B**) Analysis of aortic lesions using H&E staining (40× and 200×). (**C**–**E**) MMP-2, MMP-9, α-SMA and calponin expressions in arotas using Western blot analysis. (**F**) α-SMA and calponin expressions in arotas using IHC analysis (400×). Data were represented as means ± SD (*n*=6). **P<0.01 vs. control group. ^##^*P*<0.01 vs. HFD+agomir-NC group.

Models were injected with miR-377-3p agomir to determine the effect of miR-377-3p on AS lesions. Results of qRT-PCR analysis suggested that deletion of ApoE and HFD treatment significantly down-regulated miR-377-3p levels in aorta tissues (Figure 1A). Moreover, miR-377-3p levels in ApoE^−/−^ mice with miR-377-3p agomir were significantly higher than those in ApoE^−/−^ mice with agomir-NC (Figure 1A, *P*<0.01). H&E staining analysis of aorta tissues showed increases in intimal thickness, the area of AS plaque and fibrous caps. The reduction in AS lesions in aorta tissues was observed in ApoE^−/−^ mice with miR-377-3p agomir (Figure 1B). Additionally, HFD resulted in marked increases in MMP-2 and MMP-9 expressions (Figure 1C,D, *P*<0.01), and significant reductions in α-SMA and calponin expressions (Figure 1C,E, *P*<0.01), but these alterations were significantly reversed by miR-377-3p agomir. While α-SMA and calponin expressions in HFD+agomir-377 group exhibited an increasing tendency compared with those in HFD+agomir-NC group (Figure 1F). The results indicated that miR-377-3p was able to alleviate aortic lesions in AS mice.

### MiR-377-3p inhibits ox-LDL-induced proliferation of human VSMCs

**Figure 2:**
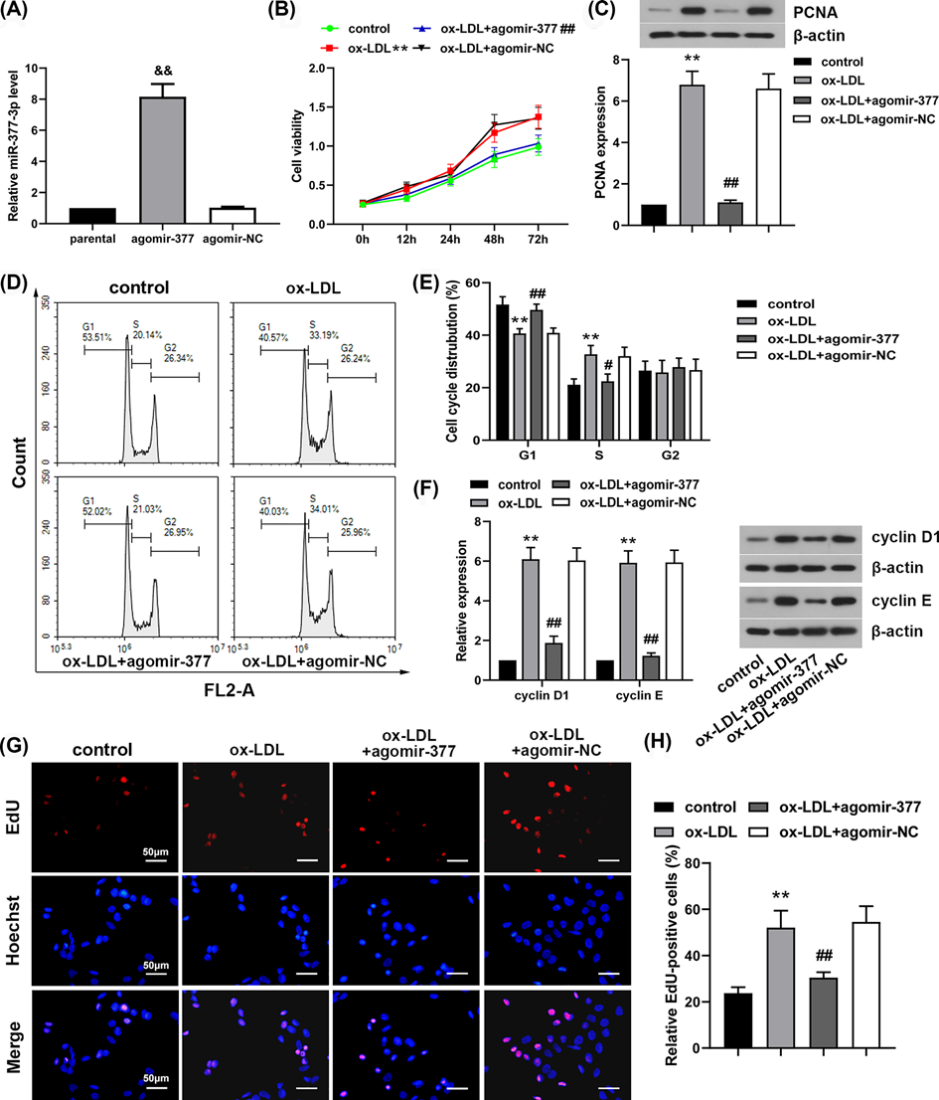
MiR-377-3p inhibits ox-LDL-induced proliferation of human VSMCs MiR-377-3p agomir or agomir-NC was transiently transfected into human VSMCs. (**A**) The mRNA level of miR-377-3p was evaluated to verify transfection efficacy using qRT-PCR after 24-h transfection. After 24-h transfection, human VSMCs were treated with 50 mg/l ox-LDL. (**B**) Cell viability using CCK-8 assay. (**C**) PCNA expression using Western blot analysis. (**D,E**) Cell cycle distribution analysis using flow cytometry. (**F**) Cyclin D1 and cyclin E expressions using Western blot analysis. (**G,H**) Analysis of S-phase cells using EdU staining (400×). Data were represented as means ± SD (*n*=3). ^&&^*P*<0.01 vs. agomir-NC group. **P<0.01 vs. control group. ^#^*P*<0.05 and ^##^*P*<0.01 vs. ox-LDL+agomir-NC group.

Human VSMCs were transfected with miR-377-3p agomir or agomir-NC. After 24 h, transfection efficacy was analyzed by qRT-PCR. Transfection with miR-377-3p agomir significantly up-regulated miR-377-3p mRNA levels in human VSMCs (Figure 2A, *P*<0.01). Then cells were treated with ox-LDL. Cell proliferation during ox-LDL treatment was analyzed using CCK-8 assay. Results showed that ox-LDL treatment significantly promoted cell viability of human VSMCs, while transfection with miR-377-3p agomir prominently reversed these changes (Figure 2B, *P*<0.01). In addition, ox-LDL treatment significantly enhanced PCNA expression, but transfection with miR-377-3p agomir significantly inhibited ox-LDL-induced expression of PCNA in human VSMCs (Figure 2C, *P*<0.01). The results of cell cycle distribution showed that ox-LDL promoted more cells to enter S phase of cell cycle (Figure 2D,E, *P*<0.01). However, transfection with miR-377-3p agomir significantly restored these alterations in cell cycle distribution (Figure 2D,E, *P*<0.01 or *P*<0.05). Besides, ox-LDL treatment up-regulated cyclin D1 and cyclin E expressions in human VSMCs (*P*<0.01, Figure 2F). Whereas cyclin D1 and cyclin E expressions in ox-LDL-treated human VSMCs were remarkably down-regulated after transfection with miR-377-3p agomir (Figure 2F, *P*<0.01). EdU assay was performed to detect cells at S phase. As shown in Figure 2G,H, ox-LDL treatment increased the percentage of EdU-positive cells, but transfection with miR-377-3p agomir significant decreased ox-LDL-induced increases in EdU-positive cells (*P*<0.01). The results demonstrated that miR-377-3p inhibited ox-LDL-induced proliferation of human VSMCs and prevented human VSMCs to enter S phase of cell cycle.

### MiR-377-3p inhibits ox-LDL-induced migration of human VSMCs

**Figure 3:**
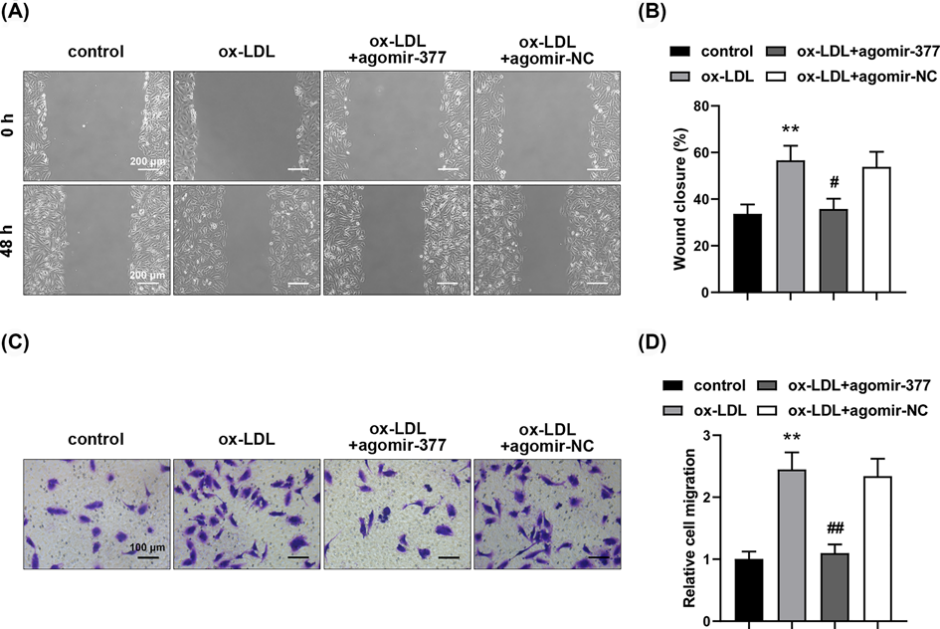
MiR-377-3p inhibits ox-LDL-induced migration of human VSMCs Analysis of cell migration using wound healing assay (**A,B**) at 0 and 48 h after wounding (100×) and transwell assay (**C,D**) after 48-h ox-LDL (50 mg/l) treatment (200×). Data were represented as means ± SD (*n*=3). **P<0.01 vs. control group. ^#^*P*<0.05 and ^##^*P*<0.01 vs. ox-LDL+agomir-NC group.

To further investigate the role of miR-377-3p in ox-LDL-induced migration of human VSMCs, wound healing assay and transwell assay were performed. The results showed that ox-LDL promoted wound closure and cell migration (Figure 3A,B, *P*<0.01). However, tranfection with miR-377-3p agomir reversed ox-LDL-induced alterations (Figure 3C,D, *P*<0.05 or *P*<0.01). The results manifested that miR-377-3p inhibited ox-LDL-induced migration of human VSMCs.

### MiR-377-3p can target NRP2 in human VSMCs

**Figure 4:**
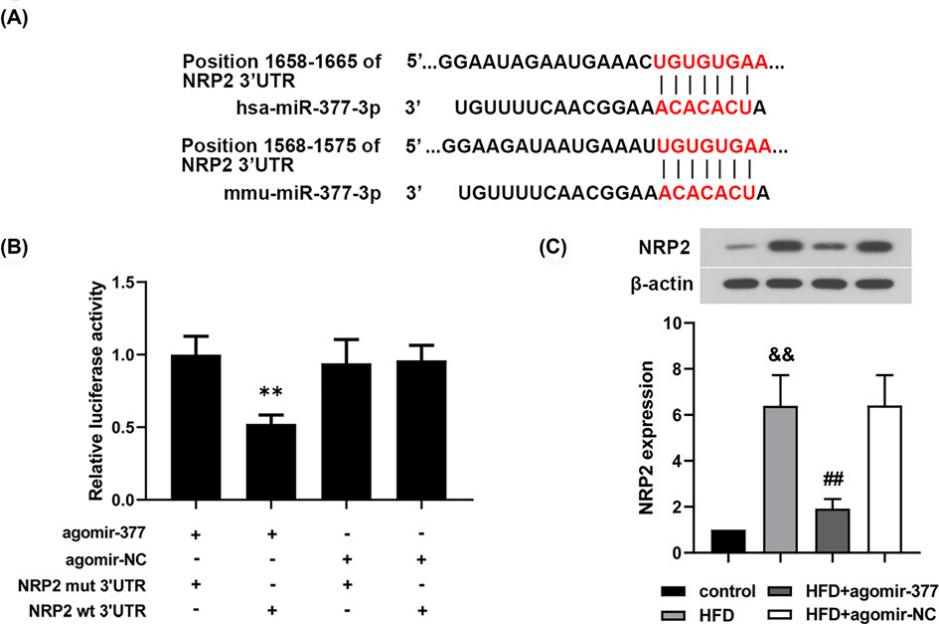
NRP2 gene is a direct target of miR-377-3p in human VSMCs (**A**) The predicted binding site between hsa-miR-377-3p/mmu-miR-377-3p and NRP2 3′UTR. (**B**) The binding between miR-377-3p and NRP2 was verified using dual luciferase reporter assay (*n*=3). (**C**) NRP2 expression in AS mice using Western blot analysis (*n*=6). Data were represented as means ± SD. **P<0.01 vs. agomir-377+NRP2 mut 3′UTR group. ^&&^*P*<0.01 vs. control group. ^##^*P*<0.01 vs. HFD+agomir-NC group.

The predicted binding site between 3′UTR of NRP2 and miR-377-3p was shown in Figure 4A. To verify whether *homo NRP2* gene was a target of hsa-miR-377-3p, the luciferase reporter plasmid containing the wt 3′UTR of NRP2 or mut 3′UTR of NRP2 was constructed. Then the luciferase reporter plasmid was co-transfected with miR-377-3p agomir or agomir-NC into human VSMCs. The co-transfection of miR-377-3p agomir and wt 3′UTR of NRP2 significantly suppressed the relative luciferase activity (Figure 4B, *P*<0.01). Additionally, it was found that the seed sequence of mmu-miR-377-3p was same to that of hsa-miR-377-3p. Moreover, *mus* NRP2 gene was also predicted as a potential target of mmu-miR-377-3p (Figure 4A). Subsequently, NRP2 expression in aorta tissues of AS mice was evaluated using Western blot analysis. Down-regulation of NRP2 expression was observed in AS mice after treatment with miR-377-3p agomir (Figure 4C, *P*<0.01). The results implied that miR-377-3p could directly target NRP2 mRNA in human VSMCs and regulate NRP2 expression in AS mice.

### MiR-377-3p inhibits ox-LDL-induced proliferation and migration of human VSMCs via targeting NRP2

**Figure 5:**
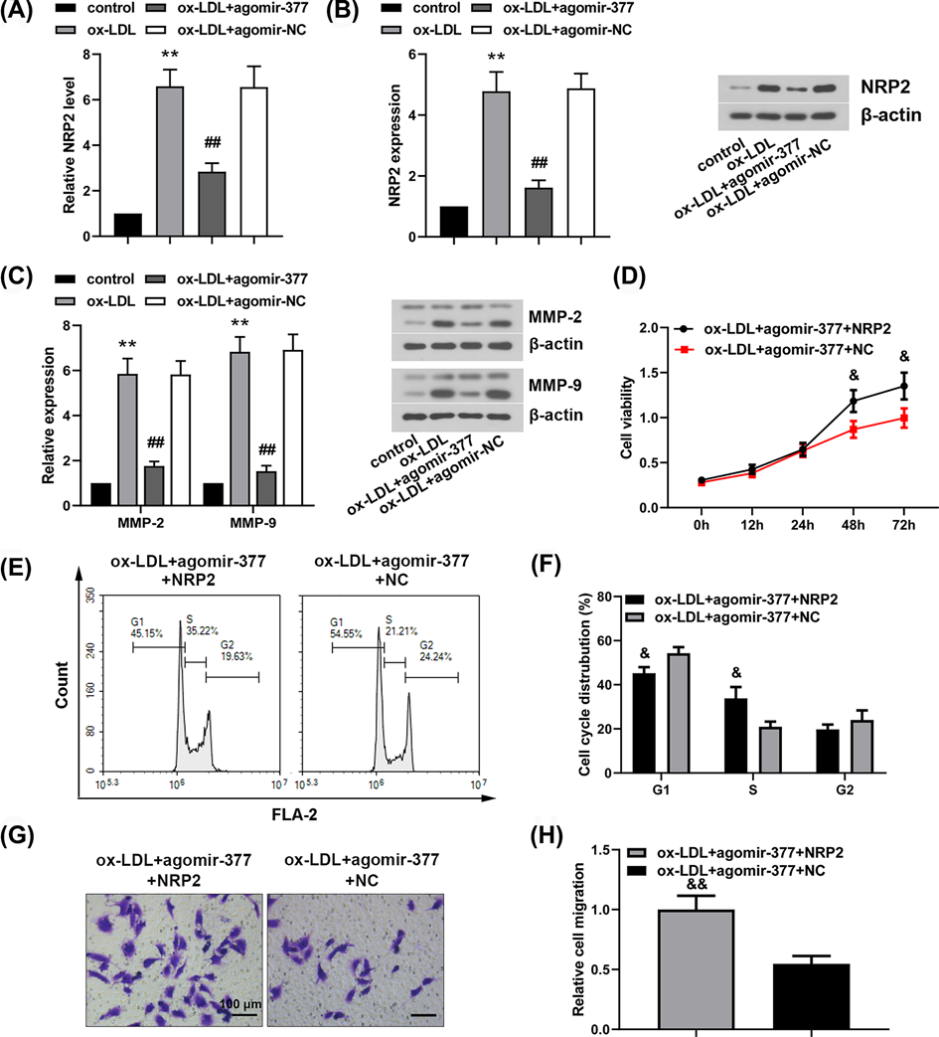
MiR-377-3p inhibits ox-LDL-induced proliferation and migration in human VSMCs via targeting NRP2 MiR-377-3p agomir was transiently transfected into human VSMCs. After 24-h transfection, human VSMCs were treated with 50 mg/l ox-LDL. (**A**) The mRNA level of NRP2 using qRT-PCR. (**B**) The expression of NRP2 using Western blot analysis. (**C**) MMP-2 and MMP-9 expressions using Western blot analysis. NRP2 overexpression vector or empty vector was co-transfected with miR-377-3p agomir in human VSMCs. (**D**) Cell viability using CCK-8 assay. (**E,F**) Cell cycle distribution using flow cytometry. (**G,H**) Analysis of cell migration using transwell assay (200×). Data were represented as means ± SD (*n*=3). **P<0.01 vs. control group. ^##^*P*<0.01 vs. ox-LDL+agomir-NC group. ^&^*P*<0.05 and ^&&^*P*<0.01 vs. ox-LDL+agomir-377 +NC group.

MiR-377-3p agomir or agomir-NC was transfected into human VSMCs. After ox-LDL treatment, the mRNA level of NRP2 in human VSMCs was evaluated by qRT-PCR analysis (Figure 5A) and Western blot analysis (Figure 5B). The results of qRT-PCR and Western blot analysis showed that miR-377-3p significantly inhibited both mRNA and protein levels of NRP2 (*P*<0.01). Subsequently, MMP-2 and MMP-9 expressions were assessed by Western blot analysis. Results showed that miR-377-3p agomir significantly inhibited ox-LDL-induced MMP-2 and MMP-9 expressions in human VSMCs (*P*<0.01, Figure 5C). In order to investigate whether miR-377-3p inhibited the proliferation and migration of human VSMCs via targeting NRP2, NRP2 overexpression vector or empty vector was co-transfected with miR-377-3p agomir into human VSMCs. After overexpression of NRP2, cell viability (Figure 5D, *P*<0.05) and cell migration (Figure 5G,H, *P*<0.01) were significantly increased, and less cells were arrested at G_1_ phase of cell cycle (Figure 5E,F, *P*<0.05). These findings indicated that overexpression of NRP2 could eliminate the effect of miR-377-3p on inhibiting human VSMC proliferation and migration. As a consequence, miR-377-3p inhibited ox-LDL-induced proliferation and migration of human VSMCs via targeting NRP2.

## Discussion

In the present study, the effect of miR-377-3p on the proliferation and migration of AS-associated VSMCs was explored *in vivo* and *in vitro. In vivo* experiments, treatment with miR-377-3p agomir effectively inhibited the progression of lesions in AS mice. *In vitro* experiments, treatment with miR-377-3p agomir was observed to inhibit cell proliferation and migration in ox-LDL-treated human VSMCs. Additionally, miR-377-3p could target the 3′UTR of NRP2 mRNA and negatively regulate the level of NRP2 in AS mice and ox-LDL-treated human VSMCs. However, NRP2 overexpression could attenuate the inhibition of cell proliferation and migration induced by miR-377-3p in ox-LDL-treated human VSMCs. Therefore, the present study illuminated that miR-377-3p inhibited AS-associated proliferation and migration in human VSMCs via targeting NRP2.

A previous study suggested that patients with hypertriglyceridemia had significantly lower miR-377 level compared with non-hypertriglyceridemic subjects and miR-377-3p might participate in regulation of triglyceride metabolism [11]. Hence, the down-regulation of miR-377-3p levels in AS mice might be related to high fat intake. MMP-2 and MMP-9 are major metalloproteinases in the development of AS plaque lesions [20]. It has been suggested that miR-377 might be used as a marker of vascular dysfunction [21]. In the study, miR-377-3p agomir was observed to decrease the area of AS lesions and down-regulate MMP-2 and MMP-9 expressions in AS mice with miR-377-3p agomir, indicating the critical role of miR-377-3p in the development of AS lesions. Similar results were found in Chen et al.’s study [11].

VSMCs are one of the major cell types that are involved in the development of atherosclerotic plaques [22]. In the pathogenesis of AS, VSMCs undergo a phenotype switch from a contractile type to a synthetic type [23]. In healthy arteries, VSMCs can secrete some contractile-related proteins including α-SMA and calponin. VSMC tansition from the contractile type to the synthetic type is characterized by low expression of contractile-related proteins [24]. VSMC phenotype transition has been reported to promote the proliferation and migration of VSMCs [14]. The abnormal proliferation and migration of VSMCs can result in the development of AS [25]. Thus up-regulation of α-SMA and calponin expression in AS mice with miR-377-3p agomir indicated that miR-377-3p might inhibit the proliferation and migration of VSMCs in AS mice. PCNA is widely used as a cell-proliferation marker protein [26]. In this study, miR-377-3p was observed to reverse ox-LDL-induced promotions of cell viablility and PCNA expressions in VSMCs. Cyclin E is required for the transition from G_1_ to S phase of cell cycle [27], while cyclin D1 is required for G_1_ phase progression [28]. In this study, miR-377-3p arrested more human VSMCs at G_1_ phase, decreased human VSMCs at S phase and down-regulated ox-LDL-induced expressions of cyclin E and cyclin D in VSMCs. These findings indicated the effect of miR-377-3p on inhibiting the proliferation of human VSMCs. The results of wound healing assay and transwell assay suggested that miR-377-3p suppressed the migration of human VSMCs. A variety of miRNAs have been demonstrated to negatively regulate the proliferation or/and migration of VSMCs, including miR-155-5p [29], let-7g [30], miR-137 [31], miR-761 [32], miR-362-3p [33] and miR-34c [34]. However, it is first reported that miR-377-3p is involved in the proliferation and migration of VSMCs.

NRP2, a non-tyrosine kinase transmembrane glycoprotein, has been confirmed as a target of multiple miRNAs, including miR-27b [35], miR-15b [36], miR-311-3p [37], miR-188 [38], miR-486-5p [39] and miR-1247 [40]. Zhu et al*.* demonstrated that NRP2 was a direct target of miR-377 in cardiomyocyte apoptosis [41]. The present study confirmed the direct binding between miR-377-3p and the 3′UTR of NRP2 mRNA in human VSMCs using dual luciferase reporter assay. NRP2 is recognized as a vascular endothelial growth factor (VEGF) receptor [42]. NRP2 can bind to VFGFC and VEGFD to regulate lymphangiogenesis [43]. Activation of NRP2 is necessary for transforming growth factor-β1-induced migration and invasion of breast cancer cells [44]. Inhibition of NRP2 enhances the migration and invasiveness of capillary ECs [45]. It has been reported that knockdown of NRP2 inhibited the migration of rat VSMCs induced by PDGF-BB and the proliferation of rat VSMCs *in vitro* and neointimal VSMCs *in vivo* [46]. In this present study, overexpression of NRP2 attenuated miR-377-3p-induced inhibition of AS-associated proliferation and migration of human VSMCs. The results implied that miR-377-3p could regulate AS-associated proliferation and migration of human VSMCs by targeting NRP2. Other miRNAs have been reported to regulate cell proliferation via targeting NRP2. MiR-1247 was able to suppress the proliferation of pancreatic cancer cells through targeting NRP2 [40]. MiR-331-3p was reported to inhibit the proliferation of cervical cancer cell by targeting NRP2 [47]. Nevertheless, miR-377-3p was reported to regulate VSMC proliferation via targeting NRP2 for the first time.

Except for VSMC proliferation and migration, miRNAs also regulate ECs and macrophages to attenuate AS development. In previous studies, various mechanisms were found to be involved in AS development. The expression of miR-126 could suppress monocyte adhesion to ECs injured by ox-LDL via targeting PI3K/Akt/NF-κB pathway [48]. MiR-590 could inhibit EC apoptosis by down-regulating TLR4/NF-κB pathway [49]. MiR-146 was found to inhibit activation of EC by targeting HuR [50]. Deficiency of miR-21 in macrophages could promote apoptosis, plaque necrosis and vascular inflammation during AS [51]. At present, whether miR-377-3p can regulate ECs and macrophages in AS progression is still unclear. Hence, further studies are required to demonstrate more details underlying miR-377-3p-induced inhibition of AS development.

## Conclusion

In summary, miR-377-3p could attenuate AS development in AS mice and ox-LDL-induced proliferation and migration of human VSMCs. Moreover, miR-377-3p exerted its effects on inhibiting VSMC proliferation and migration by targeting NRP2. The present study revealed a new role of miR-377-3p in regulating human VSMC proliferation and migration, and provided an underlying mechanism for miR-377-3p-based therapeutic prevention of AS.

## Abbreviations

agomir-NC: negative control agomir
ANOVA: analysis of variance
ApoE^−/−^: apolipoprotein E knockout
AS: atherosclerosis
CCK-8: cell counting kit-8
CVD: cardiovascular disease
EC: endothelial cell
EdU: 5-ethynyl-2′-deoxyuridine
FBS: fetal bovine serum
HFD: high-fat diet
HRP: horseradish peroxidase
H&E: Hematoxylin and Eosin
miRNA: microRNA
MMP: matrix metalloproteinase
NRP2: neuropilin2
OCT: optimal cutting temperature
ox-LDL: oxidized low-density lipoprotein
PCNA: proliferating cell nuclear antigen
PDGF-BB: platelet derived growth factor-BB
PFA: paraformaldehyde
qRT-PCR: quantitative real-time polymerase chain reaction
TLR4: toll like receptor 4
VSMC: vascular smooth muscle cell
3′UTR: 3′ untranslated region
α-SMA: α-smooth muscle actin
